# Using Virtual Reality Assisted Therapy to Reduce Cognitive Test Anxiety and Dysfunctional Metacognitions

**DOI:** 10.1192/j.eurpsy.2024.884

**Published:** 2024-08-27

**Authors:** F. Obuća, P. Ünal-Aydın, O. Aydın

**Affiliations:** ^1^Psychology, International University of Sarajevo, Sarajevo, Bosnia and Herzegovina

## Abstract

**Introduction:**

Cognitive test anxiety and dysfunctional metacognitions can significantly impact an individual’s performance and overall mental health. However, the effectiveness of various treatment strategies, including Virtual Reality (VR) therapy, is yet to be fully explored.

**Objectives:**

This study aimed to examine the effectiveness of VR therapy in reducing cognitive test anxiety and dysfunctional metacognitions in adults.

**Methods:**

A total of 64 participants were enrolled in the study, with 40 in the treatment group and 24 in the control group. Data were collected using the Metacognition Questionnaire-30, Cognitive Test Anxiety Scale, and a sociodemographic questionnaire. Paired samples t-tests were used to compare pretest and posttest scores, while independent samples t-tests were used to compare the means between the groups.

**Results:**

The findings suggest that the treatment group experienced a significant reduction in cognitive test anxiety and negative metacognition scores following VR therapy. No significant changes were observed in the control group, and there were no significant differences in pretest scores between the treatment and control groups.

**Conclusions:**

The study indicates that VR therapy may be an effective treatment strategy for reducing cognitive test anxiety and dysfunctional metacognitions. Further research is recommended to validate these findings and explore the potential of VR therapy in treating other psychological disorders.

**Disclosure of Interest:**

None Declared

